# 

**Published:** 1913-04-19

**Authors:** 


### Buildings Contemplated.

Airdrie.—The Town Council have decided to reconstruct their hospital at an estimated cost of £3,000.

Ashby.—The Leicestershire County Council have resolved to apply to the Local Government Board for authority to borrow £13,600 for a new isolation hospital.

Brandon.—The Urban District Council's surveyor has been instructed to prepare plans for the proposed new hospital.

Earsdon Grange.—A scheme to erect an isolation hospital at an estimated cost of £1,700 is under consideration.

Holywell.—The Guardians have been authorised to borrow £7,500 for a new infirmary on a site near the workhouse.

Knightwick.—Plans have been prepared for extending the Worcestershire Sanatorium for Consumptives.

Lincoln.—The Guardians have decided to rebuild their laundry and to provide new machinery at a cost estimated at £2,000.

Macclesfield.—The Hospital Committee have issued instructions for the preparation of plans for a new hospital on the cubicle system, and a nurses' home.

Newington, S.E.—A new out-patient department has been opened for the South London Hospital for Women at Newington Causeway. A freehold site of three acres, situate at Clapham Common, has been acquired for the building of the new in-patient department. The cost of the whole scheme is estimated at £55,000.

Retford.—The Town Council are considering a scheme for providing a new isolation hospital at a cost estimated at £5,710, exclusive of furnishing.

Sherbrooke, Quebec.—The Protestant Hospital Corporation propose to erect a new and up-to-date building.

### LARGE BEQUEST TO A DISPENSARY.

Mr. Harry Goddard, of Brixton Hill, left estate of the gross value of £26,272, of which the net personalty has been sworn at £19,894. With the exception of certain shares and his household and personal effects he left the residue of his property, subject to some life interests, to the Brixton, Streatham Hill, Tulse Hill, and Herne Hill Dispensary at Water Lane, Brixton.

### Books Received.

Wiley and Sons: "Chloride of Lime," by A. H. Hooker.

"Truth" Publishing Co.: "*Truth* Cautionary List, 1913."

Stanley Paul and Co.: "The Insanity of Genius," by J. F. Nisbet.

Methuen and Co., Ltd.: "Health through Diet," by K. G. Haig.

People's Refreshment House Association: "Public-House Reform."

E. and S. Livingstone: "Modern Wound Treatment," by Sir Geo. Beatson.

Edward Arnold: "Lectures on Diseases of Children," by Robert Hutchison.

Walter Scott Publishing Co.: "Digestion and Diet," by Thos. Dutton, M.D.

The University Tutorial Press: "Matriculation Directory, January 1913."

Grattan: "Pharmacy and Materia Medica for Nurses," by Horace Finnemore.

Sir Isaac Pitman and Sons, Ltd.: "Astonishing Anatomy!" by "Tingle."

Constable and Co.: "The Dentists' Register, 1913"; "The Medical Register, 1913."

Animals' Friend Society: "Cruelties in Dress," by Jessey Wade; "For Love of Beasts," by John Galsworthy.

Incorporated Sanitary Association of Scotland: "Transactions of the Incorporated Sanitary Association of Scotland, 1912."

J. Wright and Sons: "Studies in Smallpox and Vaccination," by Wm. Hanna; "Dental Anæsthetics," by W. E. Alderson.

The Scientific Press, Ltd.: "Care of Children," by R. J. Blackham; "Gynæcology for Nurses and Gynæcological Nursing" (2nd edition), by Comyns Berkeley.

Bale, Sons, and Danielsson: "Nerve Aggregation and Medical Free Paths," by H. Elliot Blake; "University Reform in Medicine and Reformed Adages," by H. Elliot Blake.

Cassell and Co.: "Diseases of Women," by G. E. Herman, M.B., and R. D. Maxwell, M.D.; "Manual of Surgical Treatment" (Vol. IV., new edition), by Cheyne and Burghard.

### Gifts and Bequests.

Mr. H. H. Wildt has contributed £105 to the general funds of the Leicester Royal Infirmary.

Mr. Bernard Hartley, of Pontefract, magistrate, has bequeathed £100 to Pontefract General Dispensary.

The City of London Hospital for Diseases of the Chest, Victoria Park, has received £63 from the Clothworkers' Company.

The Chelsea Hospital for Women has received a grant of £100 from the Clothworkers' Company towards its Rebuilding Fund.

The Great Northern Central Hospital have received £100 from the Clothworkers' Company and £50 from Colonel H. J. Hope-Edwardes.

Amongst the latest contributions received at the Bank of England for King Edward's Hospital Fund for London is one of £500 from the Clothworkers' Company.

The Rev. Thomas Sheepshanks has left £300 to the Leeds General Infirmary, £200 to the Harrogate Bath Hospital, and £100 to the Leeds General Dispensary.

The Hampstead General and North-West London Hospital has received an anonymous donation of £100 from "A Friend," and 80 guineas from the Clothworkers' Company.

The Rev. Cecil Bromley, of Maulden, Bedfordshire, has bequeathed £500 each to the Bedford County Hospital and the Yorkshire Home for Chronic and Incurable Diseases, Harrogate.

Miss Mary Dains, of Trimley House, Suffolk, has bequeathed £500 each to Dr. Barnardo's Homes, Spurgeon's Stockwell Orphanage, and the Eastern Counties Asylum for Idiots at Colchester.

Mr. Samuel Kenworthy, of Dobcross, in Saddleworth, Yorks, has bequeathed £500 each to Dr. Barnardo's Homes, £250 each to the Institute for the Blind, Bradford, and the Institute for the Blind, Oldham.

The Hon. Treasurer of the "Intermediate Homes for Incurables" acknowledges £50 from the Executors of the late Miss Frances Rainier Harris, being a legacy (free of duty) for the Mary Yolland Home for Incurable Girls, Upper Hale, Farnham, Surrey.

Mr. Murdoch McLeod, of Lytham, Lancashire, has bequeathed the residue, which will amount to about £4,500 net, after deduction of estate and legacy duty, equally between the Manchester Royal Infirmary and the Christie Hospital (Cancer Pavilion and Home).

The Grocers' Company have given £500, the Goldsmiths' Company £200, the Mercers' Company £105, and the Clothworkers' Company £105 in support of the presidency of Prince Arthur of Connaught at the centenary celebration in aid of the London Orphan Asylum, Watford.

Mr. John Thomas Haigh, senior director of Messrs. John Haigh and Sons (Lim.), of the Priestroyd Ironworks, Huddersfield, has left £1,000 to the executors of his will and his nephew, John Haigh, for distribution among such charitable institutions as they may determine; and £100 to the Huddersfield Infirmary.

Mrs. Elizabeth Akrill, of Edgbaston, has left £10,000 for the erection and endowment of a convalescent home for women and children or a home for crippled children at Clent or Rhyl, or elsewhere, as the trustees shall determine, to be known as the Akrill Convalescent Home for Women and Children or the Akrill Home for Crippled Children.

### Appointments.

Mr. R. S. Miller has been appointed fifth assistant medical officer at Colney Hatch Mental Hospital.

Mr. J. F. O'Connell has resigned his post of fifth assistant medical officer at Hanwell Mental Hospital.

Mr. David W. John, M.R.C.S., L.R.C.P. (Guy's and Cambridge), has been appointed house surgeon at the Gravesend Hospital.

The Rev. E. J. Hockley, chaplain of Hanwell Mental Hospital, has been appointed chaplain to the Horton and the Manor Mental Hospitals on the resignation of the Rev. H. de Courcy Blakeney.

Mr. F. Bolton Carter, M.S., M.D. (Lond.), F.R.C.S. (Eng.), has been elected honorary surgeon to the Leicester Royal Infirmary, to fill a vacancy caused by the retirement of Mr. C. J. Bond. Mr. Carter was appointed house surgeon to the institution in 1900, a post which he held for four years, and in 1906 he was elected honorary assistant surgeon, in which capacity he has completed seven years' service.

### Obituary.

We have to record the death in the Mission Field of two medical men—Dr. Stanley Jenkins, of Bristol, and Dr. C. F. Robertson, of London, both attached to the Baptist Missionary Society's Hospital at Sian-fu, China, as the result of having been seized with typhoid fever when working at high pressure in a crowded hospital. Dr. Robertson was unmarried, while Dr. Jenkins leaves a wife and two children.

### Personalia.

Mr. Montagu Frank Hopson, L.D.S.Eng., dental surgeon at Guy's Hospital, has been elected a member of the Board of Examiners of the R.C.S. in Dental Surgery.

The congratulations of the Council of the Royal College of Surgeons have been given to Surgeon-Major H. B. Hinton, the oldest member of the college, who attained the age of 100 on March 7 last.

An illuminated address has been presented to Mr. David Charles Lloyd-Owen, M.D., F.R.C.S.I., honorary consulting ophthalmic surgeon to the General Hospital, Birmingham, who recently resigned the post.

Mr. W. Ashburner, the first representative of Labour on the board of the Herefordshire General Hospital, has been appointed a life governor of the institution. Mr. Ashburner has been connected with the hospital for several years past.

Miss Beardoe-Grundy, lady almoner, Guy's Hospital, and Miss Grahame, lady almoner, Waterloo Hospital, are announced to have joined the "Duty and Discipline Movement," whose aim is the creation of a widespread public opinion against all forms of indiscipline. Miss Lucie Whitby, L.R.C.P. & S., who has for some time been an adherent, has sent a subscription.

Mrs. J. G. Eden, Mayoress of Ealing, who has just organised a concert on behalf of the King Edward Memorial Hospital, has received from her Majesty Queen Alexandra, through her private secretary, a letter to the effect that "she is very glad to hear that the concert has been so successful, and that you have been able to hand over so satisfactory a sum to the hospital funds." The prices were higher than is usual in Ealing, but the proceeds realised £122, the whole of which, we understand, goes to the hospital.

The triennial prize awarded by the Royal College of Surgeons, with which is awarded the John Hunter medal, has been adjudged to Dr. W. Blair Bell, of Liverpool, for his dissertation on "The Anatomy and Physiology of the Pituitary Body." The Jacksonian prize for the year 1912 has been awarded to Mr. F. W. Goyder, F.R.C.S., of Bradford, Yorks, for his dissertation on "The Embryology and Treatment of Cleft Palate." The Sir Gilbert Blane naval medals had been awarded to Fleet-Surgeon Richard Cleveland Munday, H.M.S. *Hyacinth*, 1910, and Fleet-Surgeon Edward Sutton, H.M.S. *Highflyer*, 1911, both members of the college.

